# Characterization of the complete mitochondrial genome of *Pelteobagrus intermedius* (Nichols et Pope)

**DOI:** 10.1080/23802359.2018.1443031

**Published:** 2018-02-24

**Authors:** Jia-Jia Fan, Jun-Jie Bai, Dong-Mei Ma

**Affiliations:** Key Laboratory of Tropical & Subtropical Fishery Resource Application & Cultivation, Ministry of Agriculture, Pearl River Fisheries Research Institute, Chinese Academy of Fishery Sciences, Guangzhou, China

**Keywords:** *Pelteobagrus intermedius*, Illumina sequencing, mitogenome, phylogeny analysis

## Abstract

*Pelteobagrus intermedius* is a unique freshwater fish native species in the Hainan Island and Guangxi and other water systems of China. In this study, the complete mitochondrial genome sequence of *P. intermedius* was determined from whole genome Illumina sequencing data. The total length of the mtDNA was 16,532 bp, including 13 protein-coding genes, 22 transfer RNA genes, two ribosomal RNA genes, and a non-coding control region. Phylogenetic analysis suggested that the *P. intermedius* is closely related to *Tachysurus fulvidraco*. The results will serve as a helpful reference for further studies on the conservation genetics of genus *Pelteobagrus*.

*Pelteobagrus intermedius* (Nichols et Pope) is small-scale demersal fishes. The *P. intermedius* is mainly distributed in Hainan Island and Guangxi of China (Pearl River Fisheries Research Institute, Chinese Academy of Fisheries Science 1986, 1991). In recent years, due to the serious over-arrest and the aggravating pollution of water environment, the population of *P. intermedius* has declined dramatically (Qing, Lv, Liao, et al. 2007; Qing, Lv, Zhao, et al. 2007). To conserve genetic resource, we analyzed the complete mitochondrial genome of *P. intermedius* to identify their phylogenetic position and genetic variation.

In this study, we report the complete mitochondrial genome of *P. intermedius* (KY962416). *P. intermedius* was collected from Teng County, Wuzhou City, Guangxi Province, China (E111°32′, N23°50′). Total genomic DNA was extracted from alcohol-preserved caudal fin tissue using the traditional phenol-chloroform method (Taggart et al. 1992). Leftover DNA and specimen were deposited at herbarium of Pearl River Fisheries Research Institute, Guangzhou, China, under the tag name *P. intermedius.* The genome was sequenced using Illumina-based de novo transcriptome technology and annotated using bioinformatic tools (Laslett and Canback 2008; Tamura et al. 2013). The mitochondrial genome was 16532 bp in length, which consisted of 13 protein-coding genes, 22 transfer RNA (tRNA) genes, two ribosomal RNA (rRNA) genes, and a putative control region. Except for ND6 and eight tRNA genes, which are encoded on the light strand, the remaining genes are encoded on the heavy strand. The overall nucleotide composition is A:30.15%, T:30.93%, G:14.62%, and C:24.30%, with the A + T content of 61.08%, showing an obvious anti-G bias in accordance with the mitochondrial genomes of other teleost species (Norfatimah et al. 2014; Xie et al. 2016). Among all 13 protein-coding genes, we found that most protein-coding genes for *P. intermedius* share the common initiation codon ATG, while only COXI gene which start from GTG. Besides, incomplete termination codons (T) were also found in six genes (ND2, COXII, COXIII, ND3, ND4, and Cytb), which may be completed by polyadenylation of the RNA messenger after cleavage (Nardi et al. 2001).

There are seven regions of gene overlap ranging from 1 to10 bp and 10 intergenic spacer regions ranging from 1 to 31 bp with the longest intergenic region appeared between tRNA-Asn and tRNA-Cys. Overlapped gene was believed to be associated with the transition from RNA to DNA synthesis (Hixson et al. 1986).

Similar to other mitochondrial genomes, the 12S rRNA and 16S rRNA are located between tRNA-Phe and tRNA-Leu within *P. intermedius* mitogenome, and separated by tRNA-Val. Besides, our analysis indicated that 22 tRNA genes varying from 67 to 75 bp are interspersed throughout the genome. The control region is 889 bp in length which is located between the tRNA-Pro and tRNA-Phe genes, as generally shown in most vertebrate mitochondrial genome (Liu and Yang 2013; Quan et al. 2013).

The phylogenetic tree was constructed on the basis of the complete mitogenome sequences from *P. intermedius* and other 15 closely related species in the GenBank database. According to the established phylogenetic tree, we confirm that the *P. intermedius* is much closer to *Tachysurus fulvidraco*, which coincides to the morphological taxonomy (Figure 1).

**Figure 1.**
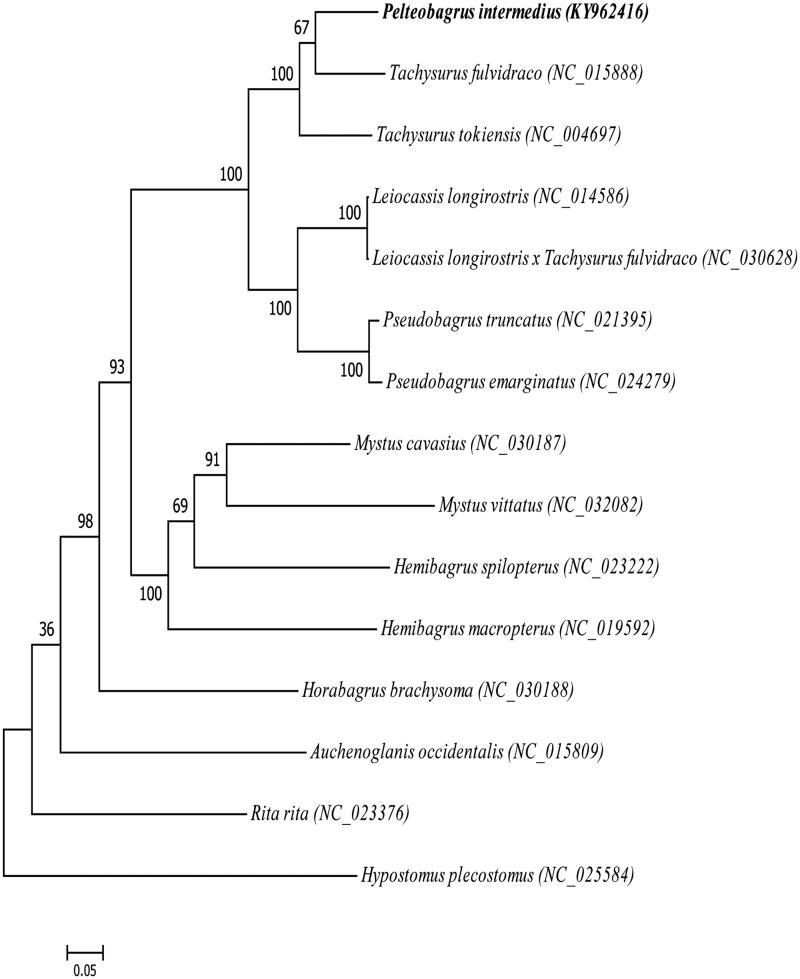
Phylogeny of 27 mitochondrial genomes with Hypostomus Plecostomus as an outgroup based on the neighbor-joining (NJ) and maximum likelihood (ML) analysis. The bootstrap values were based on 1000 resamplings.
